# Development of Radiogallium-Labeled Peptides for Platelet-Derived Growth Factor Receptor *β* (PDGFR*β*) Imaging: Influence of Different Linkers

**DOI:** 10.3390/molecules26010041

**Published:** 2020-12-23

**Authors:** Nurmaya Effendi, Kenji Mishiro, Kazuhiro Shiba, Seigo Kinuya, Kazuma Ogawa

**Affiliations:** 1 Institute for Frontier Science Initiative, Kanazawa University, Kakuma-machi, Kanazawa, Ishikawa 920-1192, Japan; nurmaya82@gmail.com (N.E.); mishiro@p.kanazawa-u.ac.jp (K.M.); 2 Faculty of Pharmacy, Universitas Muslim Indonesia, Urip Sumiharjo KM. 10, Makassar 90-231, Indonesia; 3 Advanced Science Research Center, Kanazawa University, Takara-machi 13-1, Kanazawa, Ishikawa 920-8640, Japan; shiba@med.kanazawa-u.ac.jp; 4 Department of Nuclear Medicine, Institute of Medical, Pharmaceutical and Health Sciences, Kanazawa University, Takara-machi 13-1, Kanazawa, Ishikawa 920-8641, Japan; kinuya@med.kanazawa-u.ac.jp; 5 Graduate School of Medical Sciences, Kanazawa University, Kakuma-machi, Kanazawa, Ishikawa 920-1192, Japan

**Keywords:** PDGFR*β*, peptide, imaging

## Abstract

The purpose of this study is to develop peptide-based platelet-derived growth factor receptor *β* (PDGFR*β*) imaging probes and examine the effects of several linkers, namely un-natural amino acids (D-alanine and *β*-alanine) and ethylene-glycol (EG), on the properties of Ga-DOTA-(linker)-IPLPPPRRPFFK peptides. Seven radiotracers, ^67^Ga-DOTA-(linker)-IPLPPPRRPFFK peptides, were designed, synthesized, and evaluated. The stability and cell uptake in PDGFR*β* positive peptide cells were evaluated in vitro. The biodistribution of [^67^Ga]Ga-DOTA-EG_2_-IPLPPPRRPFFK ([^67^Ga]**27**) and [^67^Ga]Ga-DOTA-EG_4_-IPLPPPRRPFFK ([^67^Ga]**28**), which were selected based on in vitro stability in murine plasma and cell uptake rates, were determined in BxPC3-*luc*-bearing nu/nu mice. Seven ^67^Ga-labeled peptides were successfully synthesized with high radiochemical yields (>85%) and purities (>99%). All evaluated radiotracers were stable in PBS (pH 7.4) at 37 °C. However, only [^67^Ga]**27** and [^67^Ga]**28** remained more than 75% after incubation in murine plasma at 37 °C for 1 h. [^67^Ga]**27** exhibited the highest BxPC3-*luc* cell uptake among the prepared radiolabeled peptides. As regards the results of the biodistribution experiments, the tumor-to-blood ratios of [^67^Ga]**27** and [^67^Ga]**28** at 1 h post-injection were 2.61 ± 0.75 and 2.05 ± 0.77, respectively. Co-injection of [^67^Ga]**27** and an excess amount of IPLPPPRRPFFK peptide as a blocking agent can significantly decrease this ratio. However, tumor accumulation was not considered sufficient. Therefore, further probe modification is required to assess tumor accumulation for in vivo imaging.

## 1. Introduction

Platelet-derived growth factor receptor beta (PDGFR*β*) is a protein that forms part of a family of transmembrane receptor tyrosine kinases [1]. PDGFR*β* is overexpressed in numerous human cancer types, including colon [2], breast [3], and pancreatic cancer [4]. The overexpression of PDGFR*β* has been associated with tumor progression features such as cell migration, metastasis, angiogenesis, and proliferation [5,6,7]. PDGFR*β* is therefore one of the preferred molecular targets for diagnosis and therapy in clinical oncology. PDGFR*β*-targeted imaging agents, which are radiolabeled probes using several types of carrier molecules with a high affinity for PDGFR*β*, such as PDGF ligand protein [8,9], aptamer [10], affibody molecules [11,12], and peptides [13,14] have been reported. Previously, we explored radioiodinated and radiobrominated quinoline derivatives as probes targeting the ATP binding site of PDGFR*β* [15,16,17]. These radiolabeled probes determined a high affinity for PDGFR*β* and a sufficient level of stability. However, the tumor accumulations of the radiolabeled probes were low, which suggests the requirement of other potential PDGFR*β*-targeted radiopharmaceuticals.

Because of their distinctive chemical and biological properties, peptides are attractive carriers when attempting to visualize a molecular target. In addition, the small molecular size of peptides compared to those of antibodies and antibody fragments mean they: can be synthesized, have easily modified structures, have high transitivity into target tissue, show fast blood clearance, and possess less immunogenicity [18,19]. Askoxylakis et al. identified linear dodecapeptide IPLPPPSRPFFK (PDGFR-P1) (IC_50_ = 1.4 μM) targeting PDGFR*β* by the biopanning technique [13]. Marr et al. then developed a PDGFR-P1 derivative, IPLPPPRRPFFK, with higher affinity for PDGFR*β* (IC_50_ = 0.48 μM) [14]. In this study, we focused on the development of the PDGFR*β*-specific peptide (IPLPPPRRPFFK)-based radiotracers with ^68^Ga, which is a promising generator produced positron emitter for positron emission tomography (PET), as PDGFR*β* imaging agents. We selected a macrocyclic ligand, 1,4,7,10-tetraazacyclododecane-1,4,7,10-tetraacetic acid (DOTA), as the chelator for ^68^Ga because it is well known that DOTA is capable of forming a stable complex with gallium [20,21,22,23]. Ga-1,4,7-triazacyclononane-1,4,7-triacetic acid (NOTA) complex has the higher stability constant than Ga-DOTA complex [24]. However, we expect that the radiolabeled PDGFR*β*-specific peptide will be the applicable to peptide receptor radionuclide therapy (PRRT) with ^90^Y, ^177^Lu, or ^225^Ac in the future. Thus, we selected DOTA instead of NOTA because DOTA is more suitable for the complexation with these therapeutic radionuclides than NOTA.

However, for some peptides in the DOTA chelation system, which is placed too close to the pharmacophore, the binding affinity may be decreased between peptides and target molecules. In this case, an appropriate spacer insertion between DOTA and the pharmacophore could improve the binding affinity [25,26,27]. Introduction of linkers can affect both the in vitro and in vivo properties of the peptide toward its molecular target and the corresponding pharmacokinetics [28]. It has been reported that hydrocarbon, un-natural amino acid, and ethylene glycol linkers display profound favorable effects in the receptor binding affinities and/or pharmacokinetics of radiolabeled peptides, such as bombesin, RGD, and α-MSH peptides [29,30,31,32].

**Figure 1:**
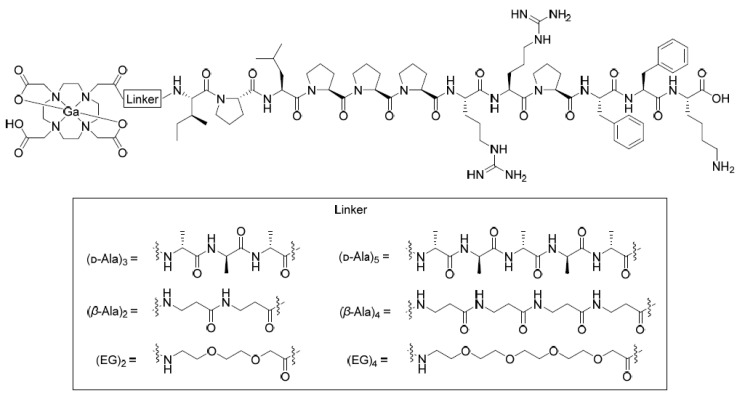
Chemical structures of Ga-DOTA-(linker)-IPLPPPRRPFFK.

In this study, peptide derivatives with ^67^Ga were synthesized to determine their viability. PDGFR*β* targeting IPLPPPRRPFFK peptide derivatives radiolabeled with easy-to-handle radioisotope ^67^Ga have a longer half-life (3.3 days) than ^68^Ga (t_1/2_ = 68 min), and therefore could serve as an alternative radionuclide for research. Moreover, to evaluate the influence of length and types of linkers on IPLPPPRRPFFK peptide properties, the linkers—namely, aaa [(D-alanine)_3_], aaaaa [(D-alanine)_5_], *ββ* [(*β*-alanine)_2_], *ββββ* [(*β*-alanine)_4_], EG_2_ [(ethylene glycol)_2_], or EG_4_ [(ethylene glycol)_4_]—were inserted between the IPLPPPRRPFFK peptide *N*-terminus and the Ga-DOTA complex (Figure 1). These linkers have been often used between radiolabeling sites and lead compounds to maintain affinity to targeting receptors because they are: uncharged (electrically neutral), highly stable against enzymatic degradation, and not sterically hindered to preserve the original bioactivity of the pharmacophore [33,34,35]. Both in vitro and in vivo properties of the radiolabeled IPLPPPRRPFFK derivatives were evaluated.

## 2. Results

### 2.1. Synthesis of Precursors and Non-Radiolabeled Compounds

**Scheme 1:**
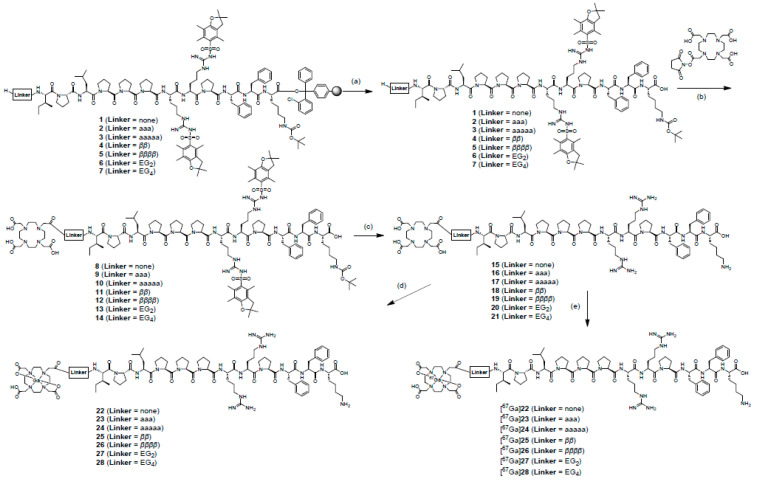
Syntheses of [^nat/67^Ga]Ga-DOTA-(linker)-IPLPPPRRFFFK (linker = none, aaa, aaaaa, *ββ*, *ββββ*, EG_2_, EG_4_). Reagents and conditions: (a) 30% HFIP, rt, 5 min; (b) DIPEA, DMF, rt, overnight; (c) 95% TFA, 2.5% triisopropylsilane, 2.5% H_2_O, rt, 2 h; (d) Ga(NO_3_)_3_, H_2_O, 40 °C, 4 h; (e) [^67^Ga]GaCl_3_, 2 M HEPES pH 5.0, 85 °C, 10 min.

A series of precursors, DOTA-(linker)-IPLPPPRRPFFK (linker = none, aaa, aaaaa, *ββ*, *ββββ*, EG_2_, EG_4_), were manually prepared using solid-phase peptide synthesis methods with traditional Fmoc chemistry (Scheme 1). The resin-cleaved protected peptides, H-(linker)-IPLPPPR(Pbf)R(Pbf)FFK(Boc), were conjugated with the chelating scaffold DOTA-NHS-ester followed by overall deprotection processes to yield the DOTA-(linker)-IPLPPPRRPFFK conjugates in ∼ 45% yields after RP-HPLC purification. The complexes, ^nat^Ga-DOTA-(linker)-IPLPPPRRPFFK, were prepared by the reaction between the precursors and gallium nitrate [Ga(NO_3_)_3_] at 40 °C for 4 h. After RP-HPLC purification, the yields of non-radioactive gallium complexes compounds were 75%, 85%, 80%, 56%, 58%, 82%, and 81% for **22**, **23**, **24**, **25**, **26**, **27**, and **28**, respectively. The structures of the non-radioactive gallium complexes were confirmed by ESI-MS.

### 2.2. Radiolabeling with ^67^Ga

The radiochemical yields of [^67^Ga]Ga-DOTA-(linker)-IPLPPPRRPFFK (linker = none, aaa, aaaaa, *ββ*, *ββββ*, EG_2_, and EG_4_) were over 85% (Table 1). After purification using RP-HPLC, [^67^Ga]Ga-DOTA-(linker)-IPLPPPRRPFFK had a radiochemical purity of over 99%, as summarized in Table 1. The identity of [^67^Ga]Ga-DOTA-(linker)-IPLPPPRRPFFK was confirmed by comparing the retention times with those of the non-radioactive compounds. The comparison showed the same retention times as ^nat^Ga-DOTA-(linker)-IPLPPPRRPFFK in HPLC chromatograms as seen in Supplementary Figures S1–S7. Precursors and radiometal complexes can be completely separated except [^67^Ga]**25** and [^67^Ga]**26** by using the isocratic system of RP-HPLC.

### 2.3. In Vitro Stability Experiments

The radiotracers, ^67^Ga-DOTA-(linker)-IPLPPPRRPFFK peptides, after a 24 h incubation period at 37 °C in PBS pH 7.4 showed high stability wherein more than 93% of radiochemical purities as intact forms. Meanwhile, the radiochemical purities of tracers after incubation were decreased in murine plasma (Table 2).

### 2.4. Octanol-Water Partition Coefficient Experiment (log P)

Log *P* values for all of radiotracers ([^67^Ga]**22**, [^67^Ga]**23**, [^67^Ga]**24**, [^67^Ga]**25**, [^67^Ga]**26**, [^67^Ga]**27**, or [^67^Ga]**28**) were less than −4.0. The data indicate that all of synthesized radiotracers are hydrophilic.

### 2.5. In Vitro Cellular Uptake Experiments

**Figure 2:**
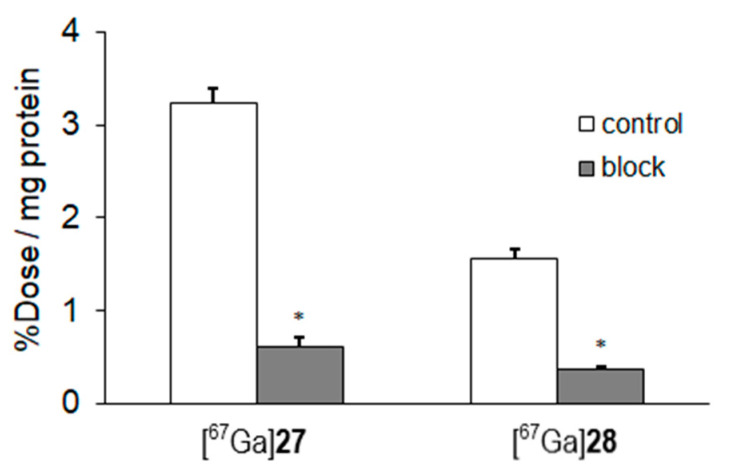
Uptake of [^67^Ga]**27** and [^67^Ga]**28** into BxPC3-*luc* cells at 1 h with or without IPLPPPRRPFFK as a blocking agent. Data were presented as mean ± SD for three samples. Significance was determined using unpaired Student’s *t*-test (* *p* < 0.05, vs. control).

In vitro cellular uptake study could be an index for the binding affinity of radiolabeled compounds to PDGFR*β*. Table 3 shows the cellular uptake results of [^67^Ga]Ga-DOTA-(linker)-IPLPPPRRPFFK (linker = none, aaa, aaaaa, *ββ*, *ββββ*, EG_2_, EG_4_) toward BxPC3-*luc* cells at several observed time points. The highest uptake of [^67^Ga]**27** into BxPC3-*luc* cells was observed. Further evaluation of [^67^Ga]**27** and [^67^Ga]**28** was performed due to their higher uptake into BxPC3-*luc* cells and higher stability in murine plasma compared to other tracers. In in vitro blocking studies, uptakes of [^67^Ga]**27** and [^67^Ga]**28** in BxPC3-*luc* cells were significantly reduced by pretreatment of excess amounts of IPLPPPRRPFFK (Figure 2).

### 2.6. Biodistribution Experiments

The biodistribution of [^67^Ga]**27** at 10 min and 1 h post-injection, and [^67^Ga]**28** at 1 h post-injection is shown in Table 4. Although the tumor uptakes of [^67^Ga]**27** and [^67^Ga]**28** were not high, the tumor-blood ratios of [^67^Ga]**27** and [^67^Ga]**28** at 1 h post-injection were 2.61 and 2.05, respectively. These results are because the blood clearance of radiotracers is so fast. Moreover, the clearance from non-target tissues was also observed to be fast. At 1 h post-injection, little radioactivity in non-target tissues was seen except in the kidney.

Detailed results of the blocking studies are shown in Table 4. The blocking agent, an excess amount of IPLPPPRRPFFK peptide, affected the biodistribution of [^67^Ga]**27**. Radioactivity levels in the blood and kidney of the blocking group significantly increased compared to that in [^67^Ga]**27** without the blocking agent. This infers that presence of the peptide might inhibit the excretion of [^67^Ga]**27** from the kidney. Radioactivity in the tumor in the blocking group also increased due to the delayed blood clearance, however the tumor-to-blood ratio at 1 h post-injection was significantly decreased by the co-injection of a blocking agent.

## 3. Discussion

PDGFR*β* expression is highly restricted in normal cells and in turn is upregulated in many tumors in humans. Because of this, PDGFR*β* is one of the targets for cancer treatment and therapy. In nuclear medicine imaging, PDGFR*β* has raised considerable interest as an attractive target in numerous human cancers. Although several single photon emission computed tomography (SPECT) or PET radiotracers have been applied to quantify the amount of PDGFR*β* expression [11,12], none have been successful for clinical use.

To optimize the in vivo pharmacological properties of radiometal-based radiopharmaceuticals, a large variety of different tools, such as chelators, linkers, and bioactive agents, have been used [36]. In this study, the chelator was fixed to DOTA, with several types of linkers between peptide and DOTA introduced. Askoxylakis et al. reported that introducing tyrosine for a radiolabeling site with ^125/131^I into the N-terminal of IPLPPPRRPFFK (yIPLPPPRRPFFK; yG2) had a 16-times higher affinity for the PDGFR*β* than IPLPPPSRPFFKY (PDGFR-P1) with tyrosine at the C-terminal [13,14]. This result suggests that the C-terminal of the peptide sequence can be crucial for receptor binding. Based on this finding, DOTA was introduced into the N-terminal of the IPLPPPRRPFFK peptide via linkers.

During the in vitro stability experiments, the ^67^Ga-DOTA complex conjugated an IPLPPPRRPFFK peptide without the presence of a [^67^Ga]**22** linker, showing a radiochemical purity level of 35% at just 1 h incubation in plasma. Contradicting our original expectations, the insertion of D-alanine and *β*-alanine linkers did not improve the peptide stability levels. However, [^67^Ga]**27** and [^67^Ga]**28** with ethylene glycol linkers showed better stability levels when in plasma (Table 2). The difference of the structures among all radiotracers is only linker part. Thus, the difference of the stability in plasma could be derived from the difference of the recognition by the enzyme in plasma. The higher stability of [^67^Ga]**27** and [^67^Ga]**28** compared to other radiotracers in plasma may be enough because their blood clearance was observed as fast.

The relatively large size of the Ga-DOTA complex might hinder the affinity of IPLPPPRRPFFK to PDGFR*β*. By inserting a linker of appropriate type and length, the peptide should maintain the binding affinity of its lead compound as it is with pharmacophore to PDGFR*β*. The proper type and length of the spacer might be different for each compound. These variations suggest a need to optimize these techniques so as to better understand the linker and spacer relationships and influences. Previously, such influences have been studied using other peptide types that target specific receptors such as the GRPr [37,38] and neurotensin receptor [36]. Results from these previous studies have seen the accumulation of radiotracers in targeted tissue increased by lengthening hydrocarbon spacers, where ultimately a length of eight carbons per linker (8-aminooctanoic acid) yielded optimum results [37,38]. Meanwhile, the insertion of a four-atom hydrocarbon spacer group (*β*-alanine) restored optimal binding affinity of tracers to neurotensin receptors rather than longer spacers [36]. Notwithstanding these previous results, this study used a variation of linker lengths, in particular an eight-atom linker (*ββ*), nine-atom linkers (aaa and EG_2_), 15-atom linkers (aaaaa and EG_4_), and a 16-atom linker (*ββββ*). However, the stability and cell uptake levels of tracers did not vary substantially depending on linker length. Results of cell uptake studies exhibited that radiotracers with D-alanine or EG linkers improved the accumulation in BxPC3-*luc* cells, which highly express PDGFR*β*, compared to [^67^Ga]**22** without a linker. However, *β*-alanine linkers did not increase the accumulation (Table 3). These results might be influenced by the difficulty experienced in separating [^67^Ga]**25** and [^67^Ga]**26** from their precursors, ultimately lowering the specific radioactivity. Namely, the precursor in [^67^Ga]**25** or [^67^Ga]**26** may decrease its uptake because ^67^Ga-labeled peptide and its precursor competitively bind to PDGFR*β*.

Biodistribution experiments of [^67^Ga]**27** and [^67^Ga]**28** were conducted because of the high uptake of BxPC3-*luc* cells and good stability levels in murine plasma compared to other tracers. As with the results of biodistribution in tumor-bearing mice, [^67^Ga]**27** and [^67^Ga]**28** showed a high tumor-to-blood ratio at 1 h post-injection, with a quick clearance from almost non-target tissues. However, the tumor uptake of [^67^Ga]**27** and [^67^Ga]**28** could be not sufficient for in vivo imaging. Therefore, the structural modification to improve the tumor uptake would be necessary. For example, the dimerization of the peptide could increase the affinity for the target receptor, and would therefore increase tumor uptake [39,40,41]. Interaction between the monomeric peptide and the receptor binding site is limited. Conversely, dimeric or multimeric peptide could have multivalent interactions, namely multivalent effects toward the receptor target. These multivalent interactions, which arise from synergistic binding of ligands, can enhance the binding affinity of ligands [42,43]. Another strategy is the insertion of a longer PEG as a linker, which could delay the blood clearance rate and increase tumor uptake of the radiotracer [44,45].

## 4. Materials and Methods

### 4.1. General

[^67^Ga]GaCl_3_ was kindly provided by Nihon Mediphysics Co., Ltd. (Tokyo, Japan). 1,4,7,10-Tetraazacyclododecane-1,4,7,10-tetraacetic acid mono-*N*-hydroxysuccinimide ester (DOTA-NHS ester) was purchased from Macrocylics (Dallas, TX, USA). Fmoc-Lys(Boc)-OH, Fmoc-Phe-OH, Fmoc-Pro-OH, Fmoc-Arg(Pbf)-OH, Fmoc-Leu-OH, Fmoc-Ile-OH, Fmoc-D-Ala-OH, Fmoc-*β*-Ala-OH, and 2-chlorotrityl chloride resin were purchased from Watanabe Chemical Industries, Ltd. (Hiroshima, Japan). Fmoc-EG_2_-OH (1-(9H-fluoren-9-yl)-3-oxo-2,7,10-trioxa-4-azadodecan-12-oic acid) and Fmoc-EG_4_-OH [1-(9H-fluoren-9-yl)-3-oxo-2,7,10,13,16-pentaoxa-4-azaoctadecan-18-oic acid] were purchased from BLD Pharmatech Ltd. (Shanghai, China). 1,3-Diisopropylcarbodiimide (DIPCI) and 1-hydroxybenzotriazole hydrate (HOBt) were purchased from Kokusan Chemical Co., Ltd. (Tokyo, Japan). *N,N*-Diisopropylethylamine (DIPEA) and Bicinchoninic Acid (BCA) Protein Assay Kit were purchased from Nacalai Tesque, Inc (Kyoto, Japan). 1,1,1,3,3,3-Hexafluoro-2-propanol (HFIP) was purchased from Tokyo Chemical Industry Co., Ltd. (Tokyo, Japan). Other chemicals and solvents were reagent grade and used as received. BxPC3-*luc* pancreatic cell line was purchased from JCRB Cell Bank (Ibaraki, Japan). Electrospray ionization mass spectra (ESI-MS) was obtained with JEOL JMS-T100TD (JEOL Ltd., Tokyo, Japan). Purification was conducted using reversed-phase high-performance liquid chromatography (RP-HPLC) system (Prominence system, Shimadzu, Kyoto, Japan). The radioactivity was measured by an Auto Gamma System ARC-7010B (Hitachi, Ltd., Tokyo, Japan).

### 4.2. Synthesis of Precursors

The peptide‒chelator conjugates were synthesized manually using a standard Fmoc-based solid-phase methodology according to a previous report with a slight modification (Scheme 1) [46]. The peptide chain (IPLPPPRRPFFK) was constructed according to the cycle consisting of (**I**) 10 min of Fmoc deprotection with 20% piperidine in dimethylformamide (DMF) and (**II**) 1.5 h coupling of the Fmoc protected amino acid (2.5 equiv.) with DIPCI (2.5 equiv.) and HOBt (2.5 equiv.) in DMF. Each Fmoc deprotection and peptide coupling step was monitored by Kaiser test. The coupling reaction was repeated to obtain Ile-Pro-Leu-Pro-Pro-Pro-Arg(Pbf)-Arg(Pbf)-Pro-Phe-Phe-Lys(Boc)-Resin (**1**). The resin-bound peptide was treated with 30% HFIP in dichloromethane for 5 min to cleave the bond between the resin and the peptide chain. After filtration, the filtrate was concentrated under reduced pressure. The crude residue Ile-Pro-Leu-Pro-Pro-Pro-Arg(Pbf)-Arg(Pbf)-Pro-Phe-Phe-Lys(Boc) was used in the following reaction without purification. The peptide (1 equiv.), DOTA-NHS ester (1.5 equiv.), and DIPEA (20 equiv.) were mixed in DMF and stirred at room temperature for overnight to obtain DOTA-Ile-Pro-Leu-Pro-Pro-Pro-Arg(Pbf)-Arg(Pbf)-Pro-Phe-Phe-Lys(Boc) (**8**). The protecting groups of peptide chain (**8**) were cleaved by the treatment with a mixture of trifluoroacetic acid (TFA):water:triisopropylsilane (95:2.5:2.5). After stirring for 2 h, the reaction mixture was concentrated by nitrogen gassing.

The crude DOTA-linker-peptide was purified by RP-HPLC on Cosmosil 5C_18_-AR-II column (10 ID × 250 mm; Nacalai Tesque) at a flow rate of 4.0 mL/min with a gradient mobile phase of 40–70% methanol in water with 0.1% TFA for 20 min (gradient system). Chromatograms were obtained by monitoring the UV absorption at a wavelength of 220 nm. The fraction containing DOTA-Ile-Pro-Leu-Pro-Pro-Pro-Arg-Arg-Pro-Phe-Phe-Lys (**15**) was determined by mass spectrometry and collected. The final lyophilized peptide of **15** was obtained in 66.0% yield as white solid. Other peptides were synthesized using the same procedure as above.

DOTA-IPLPPPRRPFFK (**15**), LRMS (ESI+) calcd for C_89_H_139_N_23_O_20_ [M + H^+^]: *m/z* = 1850.1, found 1850.3, yield: 66%.

DOTA-aaa-IPLPPPRRPFFK (**16**), LRMS (ESI+) calcd for C_98_H_154_N_26_O_23_ [M + H^+^]: *m/z* = 2064.2 found 2064.6, yield: 75%.

DOTA-aaaaa-IPLPPPRRPFFK (**17**), LRMS (ESI+) calcd for C_104_H_164_N_28_O_25_ [M + H^+^]: *m/z* = 2206.2 found 2206.2, yield: 79%.

DOTA-*ββ*-IPLPPPRRPFFK (**18**), LRMS (ESI+) calcd for C_95_H_149_N_25_O_22_ [M + H^+^]: *m/z* = 1993.1 found 1993.5, yield: 78%.

DOTA-*ββββ*-IPLPPPRRPFFK (**19**), LRMS (ESI+) calcd for C_101_H_159_N_27_O_24_ [M + H^+^]: *m/z* = 2135.2 found 2135.8, yield: 58%.

DOTA-EG_2_-IPLPPPRRPFFK (**20**), LRMS (ESI+) calcd for C_95_H_150_N_24_O_23_ [M + H^+^]: *m/z* = 1996.1 found 1996.9, yield: 71%.

DOTA-EG_4_-IPLPPPRRPFFK (**21**), LRMS (ESI+) calcd for C_99_H_158_N_24_O_25_ [M + H^+^]: *m/z* = 2098.2 found 2098.6, yield: 48%.

### 4.3. Synthesis of ^nat^Ga-Complexes

^nat^Ga-DOTA-(linker)-IPLPPPRRPFFK (X = linker) (X = 0, aaa, aaaaa, *ββ*, *ββββ*, EG_2_, EG_4_) was synthesized using a method described previously with a slight modification [46]. In brief, DOTA-IPLPPPRRPFFK (**15**, **16**, **17**, **18**, **19**, **20**, or **21**) (1 equiv.) in 50 µL of water, and Ga(NO_3_)_3_ (30 equiv.) in 50 µL of water were mixed. The reaction mixture was shaken at 40 °C for 4 h. ^nat^Ga-DOTA-(linker)-IPLPPPRRPFFK was purified by RP-HPLC on Cosmosil 5C_18_-AR-II column (10 ID × 250 mm; Nacalai Tesque) at a flow rate of 4.0 mL/min with a gradient mobile phase of 40% methanol in water with 0.1% TFA to 70% methanol in water with 0.1% TFA for 20 min. Chromatograms were obtained by monitoring the UV adsorption at a wavelength of 220 nm. The fraction containing desired product was determined by mass spectrometry and collected. The solvent was removed by lyophilization to provide ^nat^Ga-DOTA-(linker)-IPLPPPRRPFFK as white solid.

^nat^Ga-DOTA-IPLPPPRRPFFK (**22**), LRMS (ESI+) calcd for C_89_H_137_GaN_23_O_20_ [M + H^+^]: *m/z* = 1918.0 found 1918.5, yield: 75%.

^nat^Ga-DOTA-aaa-IPLPPPRRPFFK (**23**), LRMS (ESI+) calcd for C_98_H_152_GaN_26_O_23_ [M + H^+^]: *m/z* = 2131.1 found 2131.6, yield: 85%.

^nat^Ga-DOTA-aaaaa-IPLPPPRRPFFK (**24**), LRMS (ESI+) calcd for C_104_H_162_GaN_28_O_25_ [M + H^+^]: *m/z* = 2273.2 found 2273.7, yield: 80%.

^nat^Ga-DOTA-*ββ*-IPLPPPRRPFFK (**25**), LRMS (ESI+) calcd for C_95_H_147_GaN_25_O_22_ [M + H^+^]: *m/z* = 2060.0 found 2060.6, yield: 56%.

^nat^Ga-DOTA-*ββββ*-IPLPPPRRPFFK (**26**), LRMS (ESI+) calcd for C_101_H_157_GaN_27_O_24_ [M + H^+^]: *m/z* = 2202.1 found 2202.6, yield: 58%.

^nat^Ga-DOTA-EG_2_-IPLPPPRRPFFK (**27**), LRMS (ESI+) calcd for C_95_H_148_GaN_24_O_23_ [M + H^+^]: *m/z* = 2063.0 found 2063.6, yield: 82%.

^nat^Ga-DOTA-EG_4_-IPLPPPRRPFFK (**28**), LRMS (ESI+) calcd for C_100_H_158_GaN_24_O_25_ [M + H^+^]: *m/z* = 2165.1 found 2165.6, yield: 81%.

### 4.4. Radiolabeling with ^67^Ga

Radiogallium complexes, [^67^Ga]Ga-DOTA-(linker)-IPLPPPRRPFFK ([^67^Ga]**22**, [^67^Ga]**23**, [^67^Ga]**24**, [^67^Ga]**25**, [^67^Ga]**26**, [^67^Ga]**27**, or [^67^Ga]**28**), were synthesized manually. In detail, an aliquot of 25 µg of DOTA-(linker)-IPLPPPRRPFFK was dissolved in 50 µL of 0.2 M HEPES buffer (pH 5.0). After adding 100 µL of [^67^Ga]GaCl_3_ solution (7.4 MBq in 0.01 M HCl), the solution was heated at 85 °C for 10 min. [^67^Ga]Ga-DOTA-(linker)-IPLPPPRRPFFK ([^67^Ga]**22**, [^67^Ga]**23**, [^67^Ga]**24**, [^67^Ga]**25**, [^67^Ga]**26**, [^67^Ga]**27**, or [^67^Ga]**28**) was purified by RP-HPLC on Cosmosil 5C_18_-AR-II column (4.6 ID × 250 mm; Nacalai Tesque) at a flow rate of 1.0 mL/min with an isocratic mobile phase of 46% methanol in water with 0.1% TFA for 20 min. Chromatograms were obtained by monitoring the UV adsorption at a wavelength of 220 nm.

### 4.5. In Vitro Stability Experiments

The stability of radiotracers, [^67^Ga]**22**, [^67^Ga]**23**, [^67^Ga]**24**, [^67^Ga]**25**, [^67^Ga]**26**, [^67^Ga]**27**, or [^67^Ga]**28**, in PBS and murine plasma, were analyzed as described previously with a slight modification [47]. Briefly, the solution of radiotracers (37 kBq/well, 50 µL) in a sealed tube containing 0.1 M PBS pH 7.4 (450 µL) was incubated at 37 °C for 24 h. At 3 and 24 h after incubation, the purity of radiotracers was analyzed by RP-HPLC. Meanwhile, for stability assay in murine plasma, radiotracers were mixed in murine plasma at a ratio of 1:10. After incubation at 37 °C for 10 min and 1 h, an equivalent amount of ice-cold acetonitrile was added. After centrifugation at 1000× *g* at 4 °C for 10 min, the supernatant was filtered through a 0.45-µm filter followed by analyzing using RP-HPLC as described above. Then, the radiochemical purities were determined.

### 4.6. Octanol-Water Partition Coefficient Experiment (log P)

The octanol-water partition coefficients for radiotracers were determined via the assessment of their distribution in *n*-octanol and PBS (pH 7.4) using shake-flask method as described previously [48]. Radioactivity of each layer was measured using auto well gamma counter (n = 4).

### 4.7. In Vitro Cellular Uptake Experiments

BxPC3-*luc* was cultured in RPMI 1640 medium containing 10% fetal bovine serum (FBS) on 6-well culture plates (containing 1 × 10^6^ cells/well) for 24 h using a humidified atmosphere (5% CO_2_) incubator at 37 °C. After removal of medium, a solution of [^67^Ga]**22**, [^67^Ga]**23**, [^67^Ga]**24**, [^67^Ga]**25**, [^67^Ga]**26**, [^67^Ga]**27**, or [^67^Ga]**28** (7.4 kBq/well) in medium without FBS was added. After incubation for 0.5, 1, 2, and 4 h, the medium from each well was removed and the cells were washed twice with ice-cold PBS (1 mL). The cells were lysed using 1 M NaOH aqueous solution (1 mL). Its radioactivity was determined using an auto well gamma counter. The protein amount of cells was quantified using a BCA Protein Assay Kit following the manufacturer’s protocol. In detail, to a sample and a fresh set of standard solution, BSA (bovine serum albumin), in the 0.01–1 μg/mL range (25 μL) were added 200 μL of working reagent (a mixture of 50 portions of reagent A and 1 portion of reagent B) in a 96-well plate. After incubation under stirring at 37 °C for 30 min, the absorbance was measured using a plate reader at 540 nm. The protein concentration of samples was determined from calibration plot of BSA. All data were expressed as percent dose per microgram protein (%dose/µg protein).

In blocking experiments, [^67^Ga]**27** or [^67^Ga]**28** (7.4 kBq/well) in 2 mL of medium without FBS was added to each well with or without inhibitors (IPLPPPRRPFFK with final concentration 10 µM). After incubation for 1 h, radioactivity and protein concentration were determined using the same method above-mentioned.

### 4.8. Biodistribution Experiments

All animal handling procedures were approved by the Kanazawa University Animal Care Committee. Experiments with animals were conducted in accordance with the Guidelines for the Care and Use of Laboratory Animals of Kanazawa University. The animals were housed with free access to food and water at 23 °C with a 12 h light/dark schedule. Four-week-old female BALB/c *nu/nu* mice (12–17 g) were purchased from Japan SLC Inc. (Hamamatsu, Japan). The tumor-bearing model was prepared by subcutaneous inoculation of 1 × 10^7^ BxPC3-*luc* cells into left shoulder of female BALB/c *nu/nu* mice. The biodistribution experiment was performed approximately 4–5 weeks post-inoculation.

A saline solution of [^67^Ga]**27** or [^67^Ga]**28** (74 kBq, 100 µL) was injected intravenously into the tail vein of the mice. The mice were sacrificed at 10 min post-injection for [^67^Ga]**27** and 1 h post-injection for [^67^Ga]**27** or [^67^Ga]**28**. For in vivo blocking studies, 100 μL of a mixed saline solution of [^67^Ga]**27** (74 kBq) and IPLPPPRRPFFK peptide (1 mg/mouse) was injected via tile vein into the tumor-bearing mice. The mice were sacrificed at 1 h post-injection. Tissues of interest were removed and weighed. The radioactivity of the tissues was determined using an auto well gamma counter, and counts were corrected for background radiation and physical decay during counting. The data were expressed as percent injected dose per gram tissue (%ID/g).

### 4.9. Statistical Evaluation

All data were statistically analyzed using GraphPad Prism 5.0 (GraphPad Software, San Diego, CA, USA) and displayed as mean ± standard deviation (SD). The significance of in vitro and in vivo blocking studies, as well as biodistribution comparison between [^67^Ga]**27** and [^67^Ga]**28** groups was determined using Student’s *t*-test (unpaired, two-tailed). Results were considered statistically significant at *p* < 0.05.

## 5. Conclusions

In this study, we prepared seven radiolabeled IPLPPPRRPFFK peptide-based probes with different lengths and types of linkers in order to visualize PDGFR*β* expression. [^67^Ga]**27** and [^67^Ga]**28** with EG linkers exhibited better stability in murine plasma and cell uptake levels compared to other synthesized radiotracers. [^67^Ga]**27** and [^67^Ga]**28** showed high tumor-to-blood ratio at 1 h post-injection and fast clearance from most non-target tissues in the biodistribution experiments in tumor-bearing mice. However, further structural modification to increase the accumulation of the tracer in the PDGFR*β*-positive tumors is necessary for effective in vivo imaging.

## Figures and Tables

**Table 1:** Quality control results for [^67^Ga]Ga-DOTA-(linker)-IPLPPPRRPFFK.

Radiopeptides	Physical Properties		
	RC Yield	RC Purity	*t*_R_ (min)
[^67^Ga]Ga-DOTA-IPLPPPRRPFFK ([^67^Ga]**22**)	98.0%	99.3%	8.5
[^67^Ga]Ga-DOTA-aaa-IPLPPPRRPFFK ([^67^Ga]**23**)	86.6%	99.5%	11.3
[^67^Ga]Ga-DOTA-aaaaa-IPLPPPRRPFFK ([^67^Ga]**24**)	94.7%	99.3%	12.0
[^67^Ga]Ga-DOTA-*ββ*-IPLPPPRRPFFK ([^67^Ga]**25**)	97.4%	99.1%	9.8
[^67^Ga]Ga-DOTA-*ββββ*-IPLPPPRRPFFK ([^67^Ga]**26**)	97.9%	99.6%	9.6
[^67^Ga]Ga-DOTA-EG_2_-IPLPPPRRPFFK ([^67^Ga]**27**)	98.1%	99.5%	12.2
[^67^Ga]Ga-DOTA-EG_4_-IPLPPPRRPFFK ([^67^Ga]**28**)	96.7%	99.4%	12.7

HPLC system: Cosmosil 5C_18_-AR-II column (4.6 ID × 250 mm; Nacalai Tesque) at a flow rate of 1.0 mL/min with a gradient mobile phase of 45–65% methanol in water with 0.1% TFA for 20 min, with UV detector at 220 nm wavelength. RC means radiochemical.

**Table 2:** In vitro stability of [^67^Ga]Ga-DOTA-(linker)-IPLPPPRRPFFK in PBS pH 7.4 and murine plasma.

Radiopeptides	In Vitro Stability			
	In PBS pH 7.4		In Murine Plasma	
	3 h	24 h	10 min	1 h
[^67^Ga]Ga-DOTA-IPLPPPRRPFFK ([^67^Ga]**22**)	95.3 ± 2.7%	94.1 ± 0.6%	ND	35.2 ± 1.7%
[^67^Ga]Ga-DOTA-aaa-IPLPPPRRPFFK ([^67^Ga]**23**)	95.9 ± 0.7%	94.0 ± 0.5%	ND	15.7 ± 2.9%
[^67^Ga]Ga-DOTA-aaaaa-IPLPPPRRPFFK ([^67^Ga]**24**)	96.3 ± 1.1%	94.0 ± 0.5%	ND	41.4 ± 2.5%
[^67^Ga]Ga-DOTA-*ββ*-IPLPPPRRPFFK ([^67^Ga]**25**)	94.2 ± 1.7%	93.0 ± 0.4%	ND	26.2 ± 6.7%
[^67^Ga]Ga-DOTA-*ββββ*-IPLPPPRRPFFK ([^67^Ga]**26**)	96.9 ± 0.3%	95.4 ± 0.2%	ND	33.1 ± 5.7%
[^67^Ga]Ga-DOTA-EG_2_-IPLPPPRRPFFK ([^67^Ga]**27**)	96.9 ± 0.5%	95.6 ± 0.4%	84.4 ± 2.0%	75.9 ± 1.2%
[^67^Ga]Ga-DOTA-EG_4_-IPLPPPRRPFFK ([^67^Ga]**28**)	96.9 ± 0.2%	94.3 ± 0.3%	96.2 ± 1.1%	80.1 ± 0.8%

Expressed as percentage of remained intact of radiotracer. Data were presented as the mean (SD) for three samples. ND: not determined.

**Table 3:** Comparison of the cellular uptake of [^67^Ga]Ga-DOTA-(linker)-IPLPPPRRPFFK (linker = none, aaa, aaaaa, *ββ*, *ββββ*, EG_2_, EG_4_) into BxPC3-*luc* cells.

Radiopeptides	Time Points			
	0.5 h	1 h	2 h	4 h
[^67^Ga]Ga-DOTA-IPLPPPRRPFFK ([^67^Ga]**22**)	0.22 (0.03)	0.86 (0.24)	0.44 (0.10)	0.83 (0.16)
[^67^Ga]Ga-DOTA-aaa-IPLPPPRRPFFK ([^67^Ga]**23**)	1.40 (0.45)	2.07 (0.11)	1.89 (0.16)	1.07 (0.06)
[^67^Ga]Ga-DOTA-aaaaa-IPLPPPRRPFFK ([^67^Ga]**24**)	1.40 (0.27)	1.85 (0.21)	2.55 (0.08)	1.28 (0.05)
[^67^Ga]Ga-DOTA-*ββ*-IPLPPPRRPFFK ([^67^Ga]**25**)	0.49 (0.18)	0.44 (0.05)	0.51 (0.09)	0.69 (0.13)
[^67^Ga]Ga-DOTA-*ββββ*-IPLPPPRRPFFK ([^67^Ga]**26**)	0.49 (0.13)	0.62 (0.10)	0.61 (0.22)	0.53 (0.02)
[^67^Ga]Ga-DOTA-EG_2_-IPLPPPRRPFFK ([^67^Ga]**27**)	3.09 (0.50)	2.98 (0.29)	3.70 (0.16)	3.35 (0.06)
[^67^Ga]Ga-DOTA-EG_4_-IPLPPPRRPFFK ([^67^Ga]**28**)	1.29 (0.08)	1.37 (0.13)	1.09 (0.18)	1.72 (0.16)

Expressed as % dose/mg protein. Data were presented as the mean (SD) for four samples.

**Table 4:** Biodistribution of [^67^Ga]Ga-DOTA-EG_2_-IPLPPPRRPFFK ([^67^Ga]**27**) at 10 min and 1 h post-injection and [^67^Ga]Ga-DOTA-EG_4_-IPLPPPRRPFFK ([^67^Ga]**28**) at 1 h after i.v. injection in BxPC3-*luc* tumor-bearing mice.

Tissues	[^67^Ga]27				[^67^Ga]28	
	10 min	1 h	Blocking (1 h)		1 h	
Blood	3.87 (0.23)	0.15 (0.01)	0.93 (0.49)	*	0.12 (0.01)	*
Liver	0.90 (0.01)	0.22 (0.06)	0.27 (0.04)		0.20 (0.02)	
Kidney	13.88 (0.49)	3.07 (0.55)	12.17 (1.04)	*	2.96 (0.13)	
Small intestine	1.16 (0.14)	0.63 (0.28)	0.66 (0.08)		0.34 (0.33)	
Large intestine	0.87 (0.18)	0.13 (0.04)	0.25 (0.01)	*	0.11 (0.01)	
Spleen	0.99 (0.06)	0.13 (0.02)	0.29 (0.02)	*	0.07 (0.01)	*
Pancreas	0.89 (0.08)	0.13 (0.07)	0.21 (0.01)		0.07 (0.01)	
Lung	3.70 (0.11)	0.46 (0.10)	0.66 (0.05)	*	0.18 (0.00)	*
Heart	1.47 (0.47)	0.07 (0.02)	0.28 (0.03)	*	0.03 (0.00)	*
Stomach ^†^	0.19 (0.05)	0.06 (0.04)	0.05 (0.00)		0.01 (0.00)	
Bone	1.62 (0.30)	0.24 (0.06)	0.21 (0.02)		0.10 (0.02)	*
Muscle	0.86 (0.08)	0.07 (0.02)	0.22 (0.04)	*	0.03 (0.00)	*
Brain	0.13 (0.05)	0.01 (0.00)	0.02 (0.00)	*	0.00 (0.00)	*
Tumor	3.11 (0.31)	0.39 (0.10)	0.96 (0.13)	*	0.25 (0.05)	*
Tumor/Blood ^‡^	0.81 (0.11)	2.61 (0.75)	1.19 (0.48)	*	2.05 (0.77)	

Data were displayed as %injected dose/gram tissue. Each value represents mean ± SD for three, four, or seven tumor-bearing mice. Significance was determined using an unpaired Student’s *t*-test (* *p* < 0.05 vs. [^67^Ga]**27** at 1 h). ^†^ presented as %ID/tissue; ^‡^ presented as tumor to organ ratio.

## Supplementary Materials

The following are available online, Figure S1: The chromatogram of (a) [^nat^Ga]**22** and [^67^Ga]**22**, Figure S2: The chromatogram of (a) [^nat^Ga]**23** and [^67^Ga]**23**, Figure S3: The chromatogram of (a) [^nat^Ga]**24** and [^67^Ga]**24**, Figure S4: The chromatogram of (a) [^nat^Ga]**25** and [^67^Ga]**25**, Figure S5: The chromatogram of (a) [^nat^Ga]**26** and [^67^Ga]**26**, Figure S6: The chromatogram of (a) [^nat^Ga]**27** and [^67^Ga]**27**, Figure S7: The chromatogram of (a) [^nat^Ga]**28** and [^67^Ga]**28**.

## Sample Availability

Samples of the synthesized compounds are available from the authors.
