# Supplementary material for: Development of Radiogallium-Labeled Peptides for Platelet-Derived Growth Factor Receptor β (PDGFRβ) Imaging: Influence of Different Linkers
Source: Molecules. 2020 Dec 23;26(1):41. doi: 10.3390/molecules26010041 (PMC7795354; doi:10.3390/molecules26010041)
Supplement: Supplementary file 1 [file molecules-26-00041-s001.pdf]

Supplementary Materials

Development of radiogallium-labeled peptides for platelet-derived growth factor receptor *β* (PDGFR*β*) imaging: influence of different linkers

Nurmaya Effendi [1], Kenji Mishiro [2], Kazuhiro Shiba [3], Seigo Kinuya [4], and Kazuma Ogawa [5*]

[1] Institute for Frontier Science Initiative, Kanazawa University, Kakuma-machi, Kanazawa, Ishikawa 920-1192, Japan; Faculty of Pharmacy, Universitas Muslim Indonesia, Urip Sumiharjo KM. 10, Makassar 90-231, Indonesia; nurmaya82@gmail.com
[2] Institute for Frontier Science Initiative, Kanazawa University, Kakuma-machi, Kanazawa, Ishikawa 920-1192, Japan; mishiro@p.kanazawa-u.ac.jp
[3] Advanced Science Research Center, Kanazawa University, Takara-machi 13-1, Kanazawa, Ishikawa 920-8640, Japan; shiba@med.kanazawa-u.ac.jp
[4] Department of Nuclear Medicine, Institute of Medical, Pharmaceutical and Health Sciences, Kanazawa University, Takara-machi 13-1, Kanazawa, Ishikawa 920-8641, Japan; kinuya@med.kanazawa-u.ac.jp
[5] Institute for Frontier Science Initiative, Kanazawa University, Kakuma-machi, Kanazawa, Ishikawa 920-1192, Japan; Graduate School of Medical Sciences, Kanazawa University, Kakuma-machi, Kanazawa, Ishikawa 920-1192, Japan; kogawa@p.kanazawa-u.ac.jp

[*]Correspondence: kogawa@p.kanazawa-u.ac.jp; Tel.: +81-76-234-4460 (F.L.)

Contents:

Figure S1. HPLC chromatograms of [^{nat}Ga]**22** and [^{67}Ga]**22**;

Figure S2. HPLC chromatograms of [^{nat}Ga]**23** and [^{67}Ga]**23**;

Figure S3. HPLC chromatograms of [^{nat}Ga]**24** and [^{67}Ga]**24**;

Figure S4. HPLC chromatograms of [^{nat}Ga]**25** and [^{67}Ga]**25**;

Figure S5. HPLC chromatograms of [^{nat}Ga]**26** and [^{67}Ga]**26**;

Figure S6. HPLC chromatograms of [^{nat}Ga]**27** and [^{67}Ga]**27**;

Figure S7. HPLC chromatograms of [^{nat}Ga]**28** and [^{67}Ga]**28**.

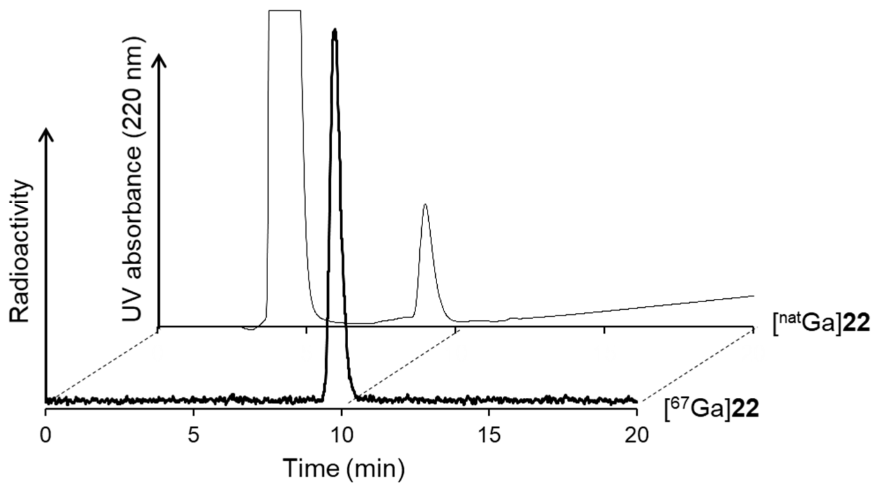

Figure S1. HPLC chromatograms of [^{nat}Ga]**22** and [^{67}Ga]**22**

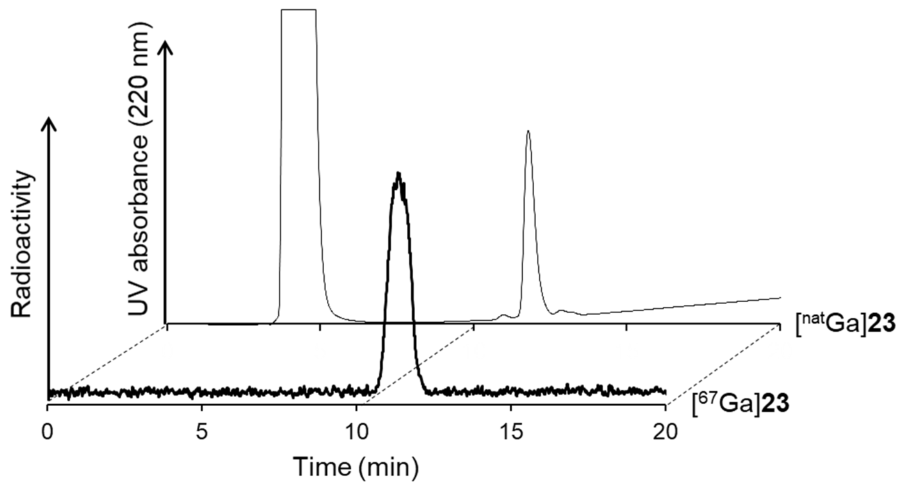

Figure S2. HPLC chromatograms of [^{nat}Ga]**23** and [^{67}Ga]**23**

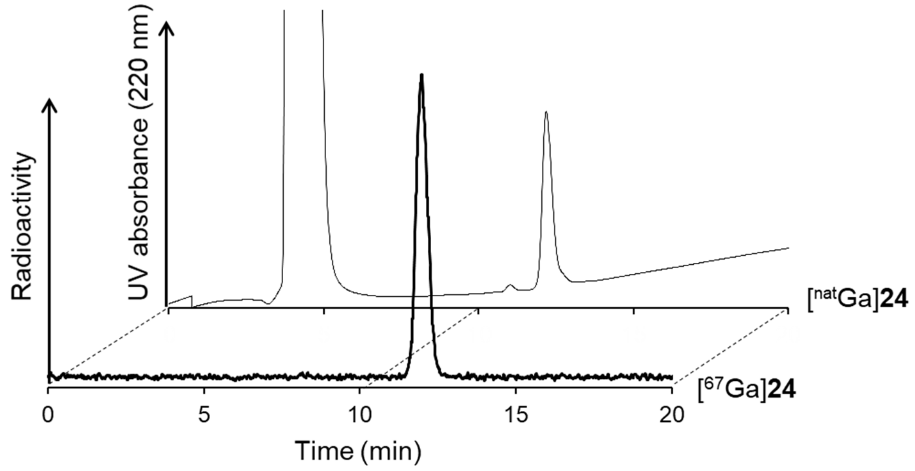

Figure S3. HPLC chromatograms of [^{nat}Ga]**24** and [^{67}Ga]**24**

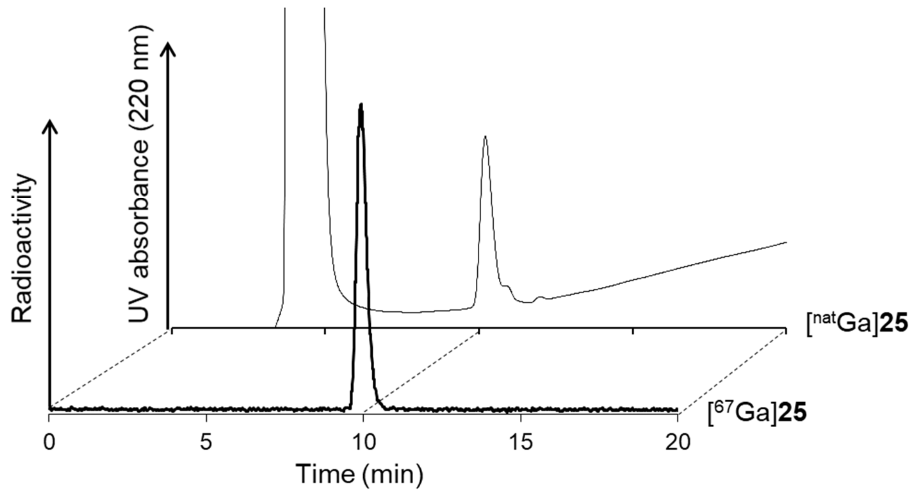

Figure S4. HPLC chromatograms of [^{nat}Ga]**25** and [^{67}Ga]**25**

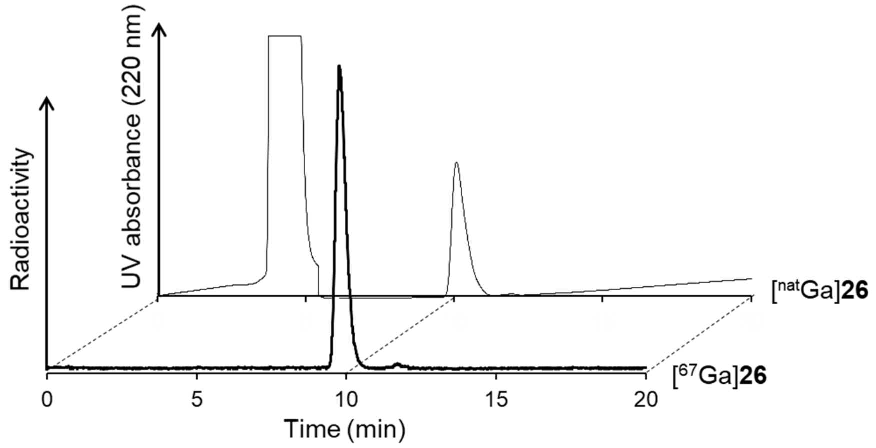

Figure S5. HPLC chromatograms of [^{nat}Ga]**26** and [^{67}Ga]**26**

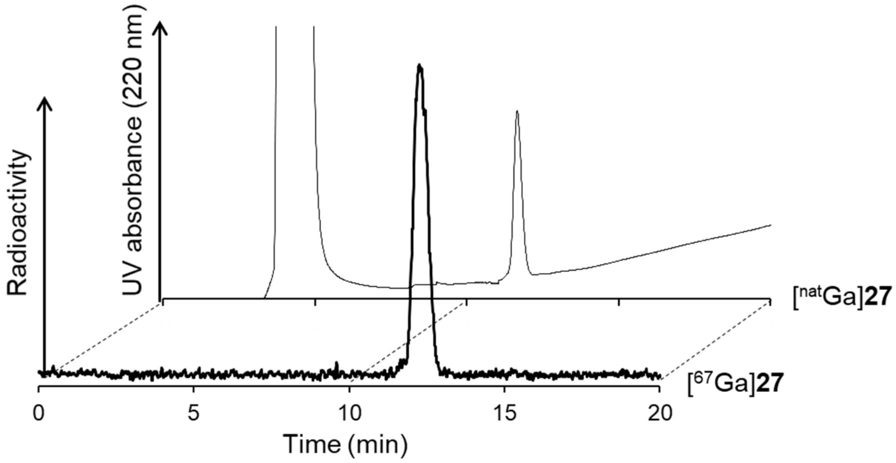

Figure S6. HPLC chromatograms of [^{nat}Ga]**27** and [^{67}Ga]**27**

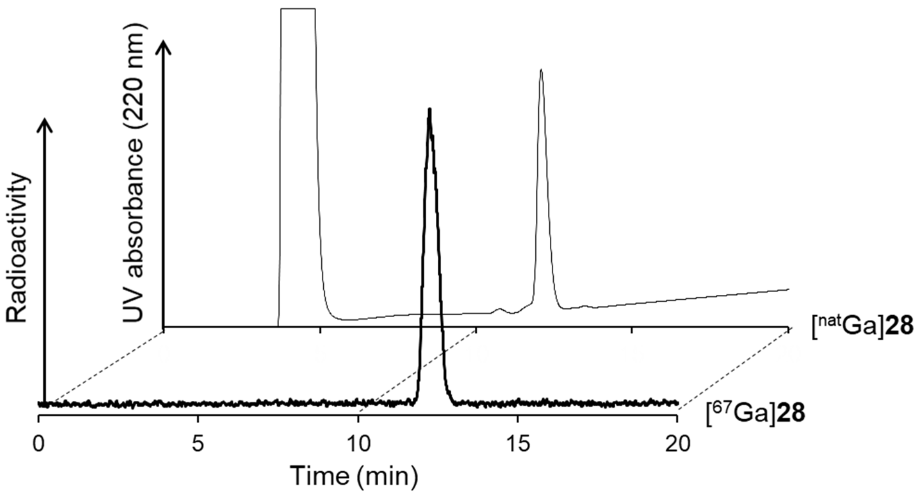

Figure S7. HPLC chromatograms of [^{nat}Ga]**28** and [^{67}Ga]**28**
